# Fabrication of Microdots Using Piezoelectric Dispensing Technique for Viscous Fluids

**DOI:** 10.3390/ma8105355

**Published:** 2015-10-14

**Authors:** Ho-Lin Tsai, Weng-Sing Hwang, Jhih-Kai Wang, Wen-Chih Peng, Shin-Hau Chen

**Affiliations:** Department of Materials Science and Engineering, National Cheng-Kung University, NO.1, Daxue Road, Tainan City 701, Taiwan; hltsai0722@gmail.com (H.-L.T.); a0936685691@gmail.com (J.-K.W.); wcpeng5028@gmail.com (W.-C.P.); den702@hotmail.com.tw (S.-H.C.)

**Keywords:** piezoelectric dispense, viscous fluid, microdot fabrication, dispensing dynamics, liquid junction

## Abstract

A simple microfluidic control method that uses a piezoelectric dispenser head is developed to fabricate microdots. A glycerol mixture was used as the test fluid to simulate conductive metallic solutions. The orifice diameter of the dispenser was 50 μm. Investigations were conducted at room temperature (25 °C). For each bipolar waveform, fluid was extruded in the form of a stretching liquid column, which eventually retracted into the dispenser orifice. Microdots were obtained by governing the liquid transfer process between the dispenser orifice and the target surface, where the gap was smaller than the maximum extrusion length during liquid column formation. Three fluid behaviors were observed using high-speed imaging, namely extrusion, impact on the target surface, and pinch-off of liquid ligament. For gaps of below 70 μm, some of the fluid sticking on the target surface resulted in a microdot diameter of 26 μm (about half of the orifice diameter).

## 1. Introduction

High-performance electronic products are essential for developing chip technology from 2-dimensional integrated circuits (ICs) to three-dimensional (3D) ICs in the microelectronics industry. 3D IC development depends on Cu-to-Cu interconnects, which allows for higher I/O, faster speed, and higher density [1]. Moreover, the feature size of Cu interconnections under development has shrunk to 10 μm. Electroplated solder bumps among microstructures on chips are believed to be best for 3D IC applications. However, low reliability due to the formation of defects and intermetallic compounds at the interactive interface between the solder and Cu has been reported [1].

In order to improve Cu-to-Cu interconnects, researchers have attempted to replace solder materials with novel nano metallic colloids and develop lower-temperature (below 250 °C) manufacturing processes. Conductive metallic colloids consist of nanoparticles, organic precursor compounds, and additives mixed in solvents. Various commercial electronic conductive inks are normally added, which exhibits a high viscosity, such as Ag-IKTS* ink (150 cP).

A high-precision fluid transfer technique is required to deliver accurate volume onto a small target surface of microstructures. Table 1 lists several fluid dispensing approaches for high-resolution applications [2,3,4,5,6,7,8,9]. All these systems require dispensing heads with a small orifice. For low-viscosity fluids (below 18 cP), ultrafine features (nanoscale to a few micrometers) have been printed using electrohydrodynamic jetting (e-jet) and aerosol jet printing systems, respectively [2,3,4,5]. For printable fluids with wide viscosity range (20 to 100,000 cP), jetting systems that combine mechanical and pneumatic forces generated by a complex micro-valve printhead have been reported [6,7]. However, these systems provide relatively low patterning ability. 

**Table 1:** Comparison of fluid dispensing approaches for high-resolution applications [2,3,4,5,6,7,8,9].

Technique	E-Jet Printer	Aerosol Jet Printing	High-Viscosity Jetting System	Piezoelectric Inkjet Printing
Dispensing components	Printhead with conducting nozzle and DC power	Ultrasonic atomizer and deposition head	Micro-valve printhead	Piezoelectric printhead
Nozzle size	0.3–200 μm	100–200 μm	50–400 μm	10–100 μm
Fluid characterization	Electrically charged fluid	Major solvent with high boiling point/low vapor pressure (compared to those of ethylene glycol)	No limitation	No limitation
Mechanism	Electrohydrodynamic	Atomization	Mechanical and pneumatic forces	Mechanical force
Recommended rheology	<18 cP 30–48 mN·m^−1^	0.7–5 cP	20–100,000 cP	< 40 cP 20–70 mN·m^−1^
Printing feature size	240 nm–210 μm	5–150 μm	10–150 μm, typically 50 μm	30–300 μm

Piezoelectric inkjet printing is a commonly used fluid dispensing technique that allows fully digital driven processing. Generally, the fluid viscosity and surface tension for piezoelectric printheads should be in the ranges of below 40 cP and 20–70 mN·m^−1^, respectively [10,11]. The ejected microdroplets depend only on the mechanical force, unlike the above-mentioned dispensing techniques. As a sufficient force propagates to the orifice, the fluid is extruded out of the orifice, forming microdroplets, whose diameter is normally 1.2–2 times that of the orifice [8,9]. Based on rheological properties of ink materials, alcohol was classified as a low viscosity of 1.07 cP [12]. In contrast, conductive polymer poly(3,4-ethylenedioxythiophene) (PEDOT) has a high viscosity of 100 cP (relative to that printable inks) [13]. With a distance of about 500 μm between the printhead and the target, spreading, receding, splashing, oscillation, and rebound occur after the droplet impacts the solid surface [14,15,16,17]. This may degrade printing quality, particularly for small targets.

Chen and Basaran [18] demonstrated that a droplet with a reduced diameter of 32 μm was ejected from a printhead with an orifice diameter of 70 μm using a specific waveform for low-viscosity mixtures of glycerol and water, ranging from pure water (1 cP) to 61 wt % glycerol (about 16 cP). Unfortunately, a similar waveform shape did not reduce the droplet diameter for conductive polymer PEDOT [13]. It is thus challenging to produce small printed dots for fluids with high viscosities. The purpose of the present study is to reduce the dot size by using a simple microfluid transfer process without a reduction in the nozzle dimensions. The effects of dynamics for a viscous glycerol mixture are observed for narrow gaps between the dispenser orifice and the target surface. The dot size can be controlled to be less than the dispenser orifice diameter. Invisible rheological influences during dot fabrication are analyzed using computational simulation.

## 2. Experimental Setup

### 2.1. Experimental Apparatus

This study uses a microfluidic control mechanism that is based on our previous studies on various droplet behaviors of ink materials, which were performed under various driving waveforms during the printing process [12,19,20]. The piezoelectric printing system (MicroFab Technologies Inc., JetLab4, Plano, TX, USA) was composed of a piezoelectric printhead and reservoir, waveform generator, observation system, and moving stages. The dynamics of fluid extrusion and transfer evolution were captured using a high-speed digital camera (NAC Image Technology Inc., Memrecam GX-3, Tokyo, Japan) with a microscope objective lens (OPTEM Zoom 125, Fairport, NY, USA). A halogen lamp (NAC, UF3252NAC, Tokyo, Japan) was used as the illumination source.

### 2.2. Preparation of Fluid

A glycerol-water mixture with 80 vol % glycerol was used as the test fluid. Fluid properties at room temperature (25 °C) were measured using a viscometer (RheoSense, Micro VISC, San Ramon, CA, USA) and a surface tensiometer (First Ten Angstroms, FTA125, Portsmouth, VA, USA). The fluid viscosity and surface tension were 72.43 cP and 66.38 mN·m^−1^, respectively. These values were confirmed using the empirical formula for viscosity reported by Cheng [21] and from the surface tension of binary solvents [22]. In addition, fluid density was estimated to be about 1.22 g·cm^−3^.

### 2.3. Experimental Conditions

**Figure 1:**
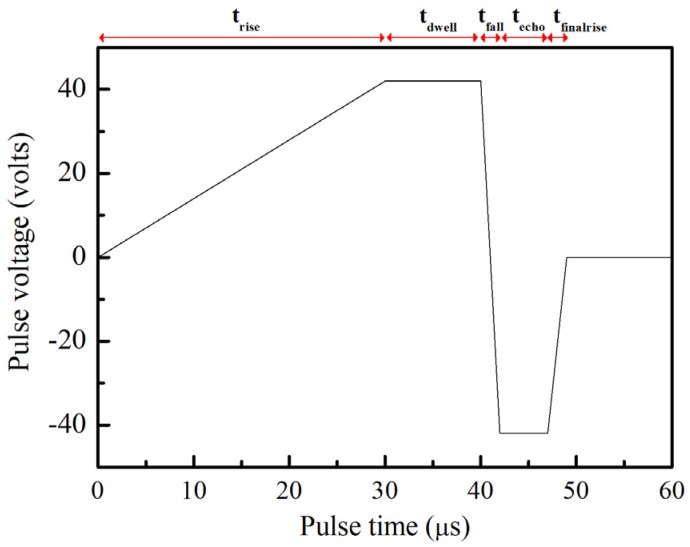
Bipolar waveform applied to glycerol solution (80 vol % glycerol) (t_rise_: 30 μs, t_dwell_: 10 μs, t_fall_: 2 μs, t_echo_: 5 μs, t_finalrise_: 2 μs, at ±42 V).

A stable backpressure can prevent the liquid from coming out of the orifice due to gravity during the dispensing process. A negative backpressure of about 1.0 bar was applied at the reservoir to control the meniscus near the orifice. A piezoelectric printhead manufactured by Microfab Technologies, Inc. with a 50 μm orifice, fixed on the *Z* axis, was employed as a dispenser head. The adjustable gap between the dispenser orifice and the target surface was set to 50, 60, 70, 80 and 90 μm. As shown in Figure 1, the dispenser was driven by a bipolar waveform sent from the waveform generator. A Cu pad was used as the target. Commercial substrates were purchased as test samples. On the substrate surface, the array pattern was constructed using electroplated pure Cu pads with a diameter of 190 μm and a thickness of 25 μm. Moreover, a protective layer of organic solderability preservative was coated onto each pad surface to prevent oxidation. The ink and substrate temperature was kept at 25 ^°^C. In order to obtain sufficient image quality of the dispensing process, a recording rate of 50,000 frames per second was used (an image was captured every 20 μs). All dispensing experiments were operated in the ambient. 

## 3. Mathematical Method

The dispensing mechanism was simulated using commercial computational fluid dynamics software, FLOW3D^®^ Version 10.0.2 (Flow Science, Inc., Santa Fe, NM, USA), as an aid to analyze the detailed physical phenomena. The core of the Flow3D code solves the continuity and momentum equations and uses an extra variable that specifies the fraction of fluid within the elements. Detailed descriptions of the simulation processes can be found elsewhere [23].

**Figure 2:**
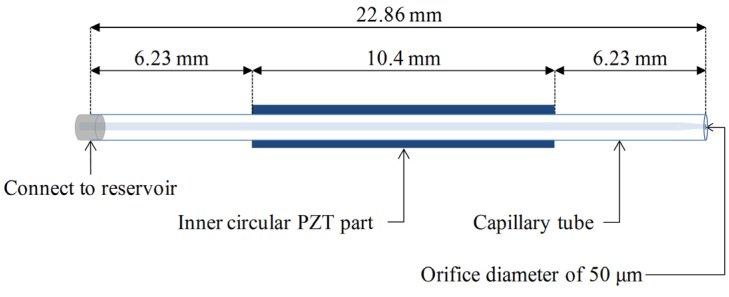
Dispenser head geometry.

The geometry of the piezoelectric dispenser head used to fabricate the microdots is shown in Figure 2. The dispenser head has a length of 22.86 mm and an orifice diameter of 50 μm. More dimensions can be found in a previous study [24]. The model of the actuated piezoelectric part was designed as a moving object in the numerical model [25]. A grid size of 3 μm × 3 μm was applied for an axisymmetric uniform mesh of 1,430,924 elements in cylindrical coordinates. The pressures were generated by the moving part and propagated toward both ends of the dispenser head, namely the fluid support end and the dispenser orifice end. For the end connected to the reservoir, a boundary condition (a specific pressure) was set to be the backpressure in the experiment (1.0 bar). The other end was the dispenser orifice (diameter: 50 μm), which allowed the fluid to be extruded outward. The test fluid (80 vol % glycerol solution) was modeled as an incompressible and Newtonian fluid.

## 4. Results and Discussion

Functional ink materials have been developed for electronics applications. Aqueous inks are widely applied due to their eco-friendly solvents, such as deionized (DI) water, ethylene glycol, and glycerol. Therefore, an aqueous glycerol mixture was employed to evaluate the dispensing process [26,27]. The viscosity of glycerol-water mixtures can be adjusted from 1 to 1000 cP via the mixing ratio [21]. Thus, this kind of fluid was useful to evaluate the dispensing process and understand the fundamental principles during manufacturing process. Meanwhile, the coffee ring issue after drying the printed patterns would not be considered. Studies have used viscous ethylene glycol and glycerol mixtures to investigate the formation of a single droplet at the optimal waveform conditions [12,26,27]. The influence of droplet formation in inkjet printheads can be analyzed in terms of the *Z* number:

Z = (D_noz_ρσ)^1/2^/μ = Oh^−1^ (1)

where D_noz_ is the nozzle diameter, σ is the surface tension, ρ is density, and μ is the viscosity. Z is equivalent to the inverse of the characteristic Ohnesorge number (Oh). A previous study [28] predicted that droplet formation in an inkjet printing system occurs in the range of *Z* > 2. When the *Z* number is near 2, a detached long ligament from the orifice becomes contracted and eventually forms several droplets when waveforms with high pulse amplitudes are applied [29]. In contrast, a longer liquid column forms when the aqueous glycerol solution is 80 vol % glycerol, with a high viscosity of 72.43 cP and a surface tension of 66.38 mN·m^−1^. Even though the equivalent *Z* number is approximately 0.88, which is outside the suitable range, microdots can be fabricated when a specific dispensing process is applied.

### 4.1. Liquid Column Formation

Figure 1 shows the waveform conditions for the test fluid. The bipolar waveform had the following parameters: t_rise_: 30 μs, t_dwell_: 10 μs, t_fall_: 2 μs, t_echo_: 5 μs, and t_finalrise_: 2 μs at pulse amplitudes of ±42 V. Pulse times control the action of the dispenser head. t_rise_ is the rising time for the electric voltage applied on the inner circular, leading zirconate titanate (PZT) part to increase from zero to a pre-designated positive voltage. Within t_rise_ in rising voltage, a negative pressure was generated by the contraction of the PZT part. In the process, t_dwell_ is the holding time at the positive pulse voltage that the contracted PZT part was fixed and the fluid was stably drawn into capillary tube. Meanwhile, t_fall_ is the transferring time from positive to a negative pulse voltage, in which a positive pressure was generated with the expansion of the PZT part. t_echo_ is shown as a time duration at the negative pulse voltage; when t_echo_ at a negative voltage, the expanded PZT part was fixed and pressure waves propagate along capillary tube. During the recovering time, the so-called t_finalrise,_ the pulse voltage raises to zero. In the final stage, the PZT part was recovered to initial state. These pulse conditions are similar to those in our previous studies, which studied the droplet formation of ethylene glycol under high pulse amplitudes [12,19].

**Figure 3:**
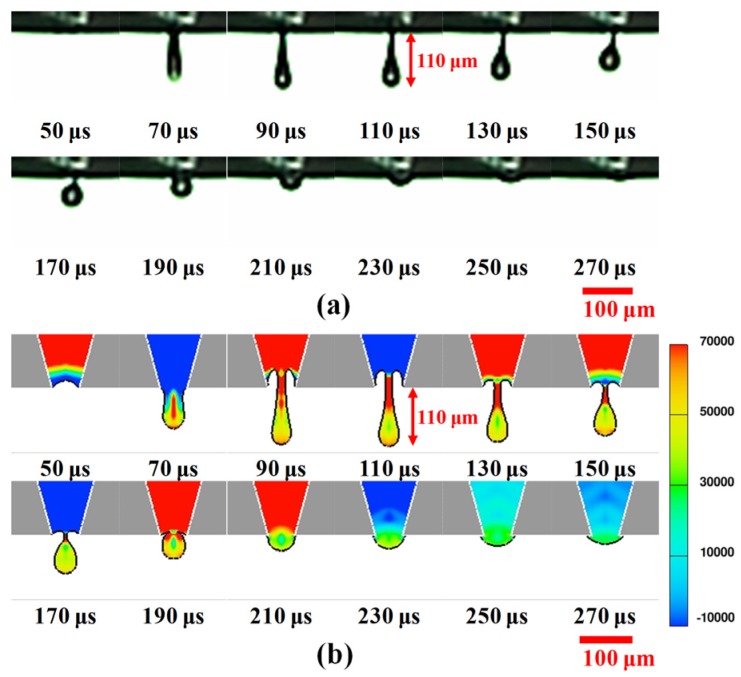
Evolution of liquid column formation with bipolar waveform using piezoelectric dispenser. (**a**) Experimental images recorded using high-speed camera and (**b**) Computed predictions of extruded fluid shapes and pressure fields at selected axial positions. Color bar represents contours of pressure distribution (unit: bar) and the red scale bars are 100 μm.

Figure 3a shows the evolution of a steady microscopic liquid column. It can be observed that the liquid column forms and retracts into the orifice in each cycle of the waveform. Between 50–70 μs, the test fluid is pushed out of the orifice and then dramatically stretches to 77 μm during the formation of a liquid column. At 90 μs, an obvious necking region forms near the orifice as the ligament elongates continuously and changes its morphology to a bulbous head. It then slightly extends to a maximum length of 110 μm at 110 μs, possibly due to reflected positive pressure. This indicates the transient balance force between inertia force and fluid properties. After 130 μs, the extruded liquid column starts to retract into the orifice, with the thinning tail thread of the necking region still attached. The entire fluid retracts into the orifice for 270 μs due to the suction effect induced by negative pressures and the fluid viscous force.

Figure 3b shows that the simulated liquid column formation is mostly consistent with the experimental observations. The colors of the scale bar represent with the same variation of pressure contours from red to blue as followed descriptions. Moreover, the meniscus is sensitive to oscillation caused by pressure variation. At 50 μs, the appearance of an inward moving meniscus is caused by a negative pressure at the orifice. Instantaneously, a positive pressure propagates through the capillary tube and then the extruded fluid rapidly grows into a ligament. At 90 μs, a cave forms inside the orifice. Moreover, the fluctuation of the cave is related to the reflected pressures. Once a negative pressure reflects to the orifice, the liquid level of the cave increases. Meanwhile, the tail part of the ligament starts to neck and thin. Between 190 and 210 μs, a reflected positive pressure pushes the cave level to a lower position, making it contact the residue fluid. Finally, entire fluid is retracted into the orifice.

### 4.2. Analysis of Pressure Distribution Along Capillary Tube

**Figure 4:**
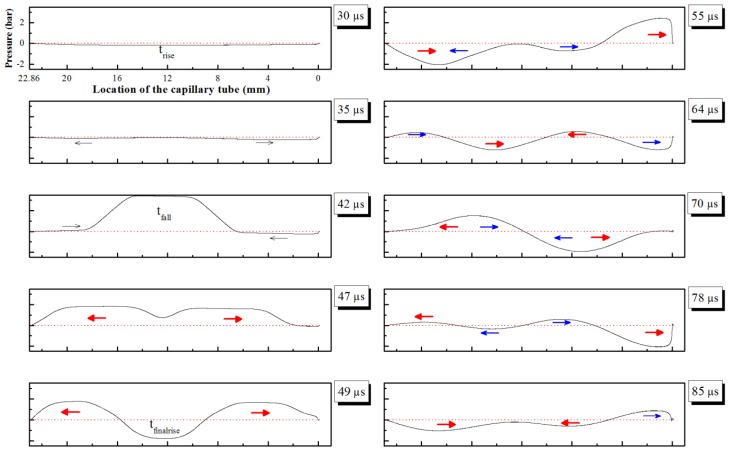
Pressure distributions versus time induced with bipolar waveform along capillary tube. Dispenser orifice is located at 0 mm and reservoir is located at 22.86 mm. Pressure propagation and reflection are denoted by black arrow for t_rise_, red arrow for t_fall_, and blue arrow for t_finalrise_.

**Figure 5:**
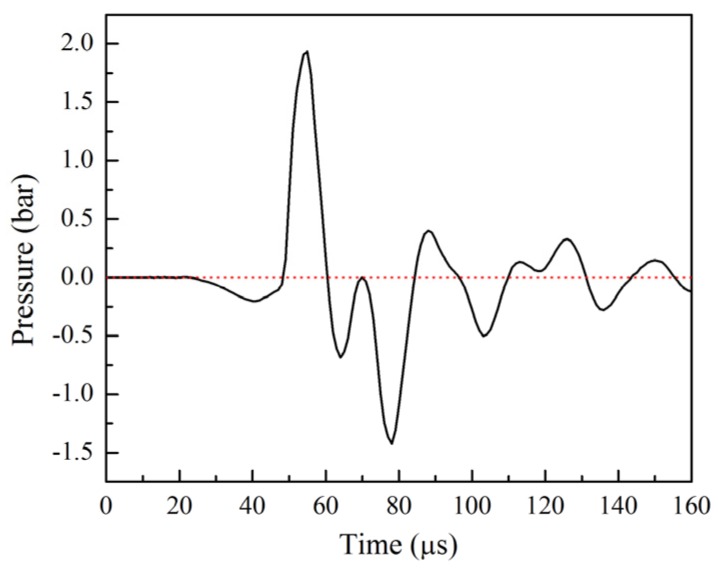
Pressure-time history calculated for dispenser outlet.

Bogy and Talke [30] showed the propagation and reflection of acoustic waves inside the capillary tube of a piezoelectric printhead. A positive pressure was produced by the expansion of the inner circular PZT part, in contrast to the negative pressure formed by the contraction. The fluid was extruded as a sufficient positive pressure propagated to the orifice end. Figure 4 shows the pressure distributions induced along the capillary tube during liquid column extrusion. Figure 5 shows the pressure-time history for the dispenser outlet.

The pressure distributions in Figure 4 are consistent with Bogy’s theory. It was assumed that the closed end is 0 mm at the orifice position of and that the open end is 22.86 mm and connected to the reservoir. A pressure wave changes sign when it reflects from an open end and it retains its sign when it reflects from a closed end [30]. In addition, the pressure profiles represent the effect of the bipolar waveform conditions. A small negative pressure was formed by t_rise_ at 30 μs, and immediately divided into two waves toward the ends. At 42 μs (corresponding to t_fall_), these two waves were reflected and a large positive pressure formed from 42 to −42 V for a short duration time of 2 μs. In order to reduce the extrusion force during t_fall_, a contraction force formed from −42 to 0 V was immediately utilized at 49 μs (corresponding to t_finalrise_).

The pressure profiles in Figure 5 show the variation of pressure for dispenser outlet. In the first stage (23–49 μs), a slight negative pressure was produced from 0 to 42 V during t_rise_. This is done to draw fluid into the capillary tube. In the next stage (t_fall_), a large positive pressure peak of about 1.9 bar appears. Partial pressure transforms into kinetic energy of the extruded fluid through the orifice, leading to a high growth rate of the liquid column. In the third stage, pulling forces are related to the negative pressure propagation during t_finalrise_ and the large reflected pressure during t_fall_ from the reservoir end (60–84 μs). At 85 μs, the reflected positive pressure during t_finalrise_ provides additional force to continuously stretch liquid column achieving maximum length of 110 μm. The residue pressures propagate along the capillary tube until the remaining energy is viscously dissipated and completely consumed.

### 4.3. Fabrication of Micro Dots

**Figure 6:**
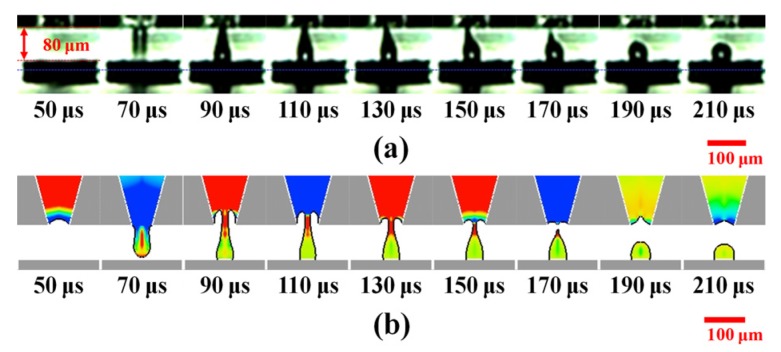
Evolution of dispensing process for gap of 80 μm. (**a**) Experimental observations and (**b**) Simulation results.

In order to generate microdots during liquid column extrusion, the gap between the orifice and the target surface must be smaller than the length of the liquid column. Figure 6a shows the dispensing dynamics for a gap of 80 μm. The ligament tip touches the target at 90 μs and then slightly spreads without further extension on the surface. Most of the extruded fluid adhered on the target surface, forming a droplet due to adhesive force from the target surface. In addition, a tail thread connected to the droplet was necking near the orifice. Until 170 μs, the thinning thread was detached. At 190 μs, the dot diameter was 48 μm and the dot height was 41 μm (smaller than the orifice diameter). In Figure 6b, the numerical simulation results show that the fluid was dispensed for a gap of 80 μm, which is consistent with the experimental results. In the period 150–170 μs, a large positive pressure appears at the tail thread, whereas a negative reflected pressure arrives near the orifice. The thinning thread cannot sustain the large pressure gradient between these regions. Therefore, the thinning thread of the tail part detaches near the orifice at 170 μs. Afterward, the reflected pressures do not further affect the dispensed fluid. The microdots were generated using a stable deposition mode due to the extrusion of the liquid column having low kinetic and viscous dissipation, unlike that for droplet ejection for low-viscosity liquids [31]. The final equilibrium stage of the dot is reached at 210 μs.

**Figure 7:**
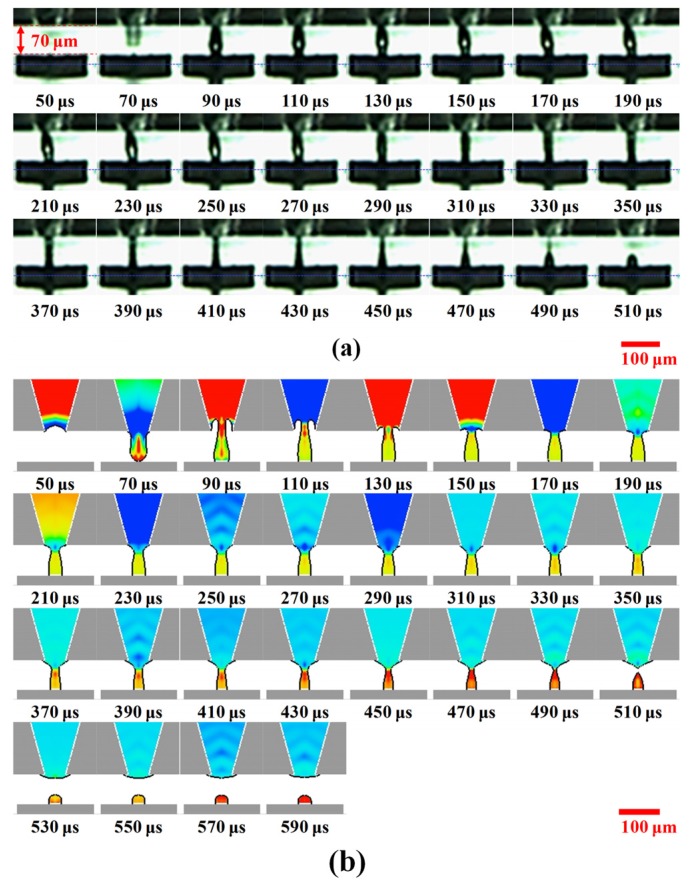
Evolution of dispensing process for gap of 70 μm. (**a**) Experimental observations and (**b**) Simulation results.

Figure 7a shows a significant difference in dynamics when the gap is decreased to 70 μm. At 90 μs, the liquid column tip impacts the target surface, and the liquid junction forms immediately. Because the limited liquid column cannot stretch its length for this gap, the leading head along the liquid junction rebounds, causes swelling in the middle region. This swelling is invisible until the extra energy is gradually depleted at 290 μs. The morphology of the liquid junction becomes a cylinder during 310–390 μs and its width decreases from 21 to 14 μm. Afterward, the liquid junction near the orifice starts to neck at 410 μs, followed by the formation of a dot connected to a thin tail thread. At 510 μs, the tail thread detachs, resulting in a dot diameter of 26 μm and a dot height of 26 μm. Dots with high contact angles of 83° and 110° were observed for dispensing gaps of 70 and 80 μm, respectively. This indicates poor wetting ability between the fluid and the target surface, which is due to a protective layer covering the Cu pad surface. The surface energy of the target surface was about 38 mN·m^−1^, and thus the surface was hydrophobic. The fluid is thus limited to a small area without extreme extension as the liquid column contacts the target surface.

Figure 7b shows the numerical simulation results for a fluid dispensed for a gap of 70 μm. At 90 μs, a cave forms inside the orifice. During 130–150 μs, the fluid is pushed out, refilling the cave, as the reflected positive pressure arrives at the orifice. The tail part near the orifice becomes broader than that for a gap of 80 μm. A liquid junction forms via the transient balance between adhesive force from the target surface and suction effect along the liquid column. In order to decrease the surface energy of the liquid junction, the fluid flows toward the opposite direction. Some of the fluid becomes stuck on the target surface, and the remaining fluid is drawn into the orifice as soon as the tailing thread forms. Meanwhile, a large pressure gradient appears at the pinch-off position, which is far away from the orifice.

### 4.4. Fluid Dispensing Mechanism

**Figure 8:**
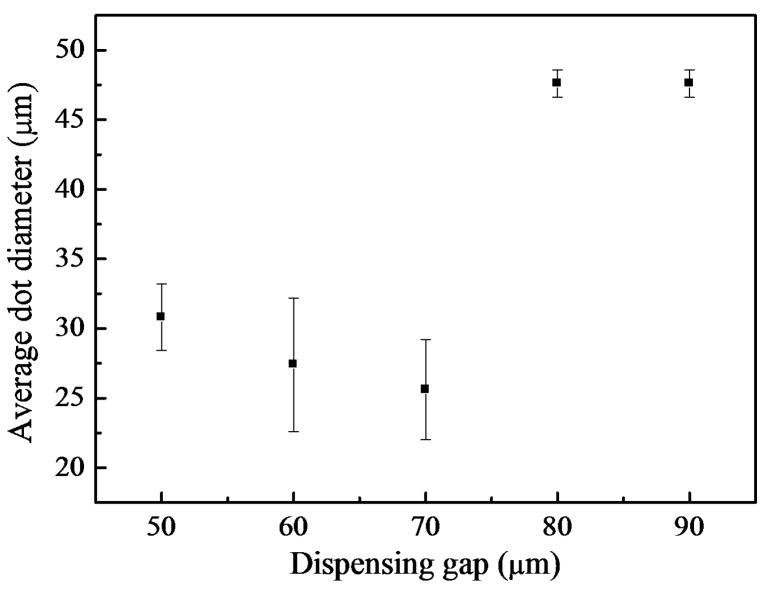
Average dot diameter versus dispensing gap for aqueous glycerol mixture (80 vol % glycerol).

Figure 8 shows the variation of the dot diameter with gap. Two dispensing models can be considered depending on the dot size. The dispensing dynamics for gaps of 80–90 μm are identical. For these gaps, the dot diameter is 48 μm and the dot volume is about 51 pL. For gaps of 60–70 μm, the average dot diameter decreases by at least 50% and the dot volume is reduced to about 5 pL. For the ultra narrow gap of 50 μm, the dot diameter slightly increased to 31 μm because the additional energy causes the wide spreading of the dot edge as the liquid column impacts the target surface.

**Figure 9:**
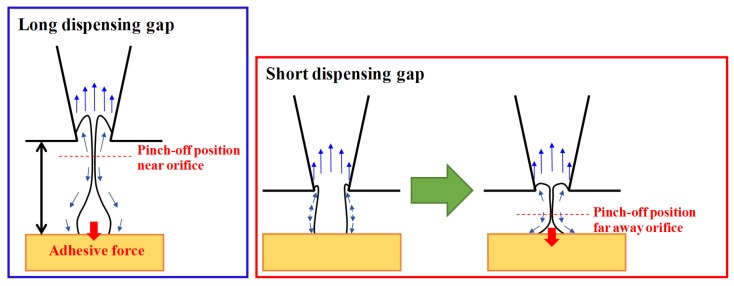
Schematic illustrations of two fluid dispensing dynamics for narrow dispensing gaps.

Yang *et al*. [7] reported a wide-viscosity jetting system that combines mechanical and pneumatic mechanisms. A high force is applied to the jetting materials. For an orifice diameter of 50 μm, the dot volume was 2–15 nL. During the process of jetting materials, a liquid column formed at the gap between the nozzle and the substrate (about 1.5 mm), similar to that formed here. Figure 9 shows the pinch-off positions of the liquid column and the characteristic times during the entire dispensing process for various gaps. Eggers [32] derived the following equation for predicting the natural length:

*l_v_* = μ^2^/ρσ (2)

where viscous and surface tension forces are balanced in the pinch-off region of the liquid thread. Some experimental studies discussed the effects of the viscous length scale during fluid dynamics of microdroplet jets [26,33]. The natural length for glycerol solution with 80 vol % glycerol is estimated to be approximately 65 μm. From experiments for gaps of 60 and 70 μm, entire dispensing time is imposed for 510 μs. Since kinetic energy of the extruded fluid cannot dissipate immediately, the leading head of liquid column rebounds along the liquid junction. In addition, this characteristic length is speculated to be caused by the transient equilibrium between the retraction force of the liquid column and the adhesive force of the substrate surface after the formation of a broad liquid junction. In contrast, large dots were fabricated for gaps of above 80 μm within 210 μs.

## 5. Conclusions 

In this paper, the reduction of dot dimensions was achieved by controlling the dispensing gap in a piezoelectric printing system. The conclusions are summarized as follows.

1. The liquid column of the aqueous glycerol solution was extruded by the lower actuation pulse obtained with a bipolar waveform.
2. The liquid column was directly detached near the orifice for the dispensing gaps of 80 and 90 μm. However, the liquid junction was formed for the dispensing gaps of 50 and 70 μm which were closed to the natural length of 65 μm.
3. Dots with a diameter of 48 μm were obtained for dispensing gaps of 80 and 90 μm.
4. Microdots with diameters half that of the orifice were fabricated with dispensing gaps of below 70 μm.
